# Validation of a Psychosocial Chronic Stress Model in the Pig Using a Multidisciplinary Approach at the Gut-Brain and Behavior Levels

**DOI:** 10.3389/fnbeh.2019.00161

**Published:** 2019-07-16

**Authors:** Sophie Menneson, Samuel Ménicot, Stéphanie Ferret-Bernard, Sylvie Guérin, Véronique Romé, Laurence Le Normand, Gwénaëlle Randuineau, Giulio Gambarota, Virginie Noirot, Pierre Etienne, Nicolas Coquery, David Val-Laillet

**Affiliations:** ^1^INRA, INSERM, Univ Rennes, Nutrition Metabolisms and Cancer, NuMeCan, Rennes, France; ^2^Phodé, Terssac, France; ^3^INSERM, LTSI – UMR 1099, Univ Rennes, Rennes, France

**Keywords:** depression, antidepressant, behavior, neuroimaging, monoamines, neurogenesis, HPA axis, microbiota

## Abstract

Psychological chronic stress is an important risk factor for major depressive disorder, of which consequences have been widely studied in rodent models. This work aimed at describing a pig model of chronic stress based on social isolation, environmental impoverishment and unpredictability. Three groups of animals of both sexes were constituted. Two were exposed to the psychosocial stressors while receiving (SF, *n* = 12) or not (SC, *n* = 22) the antidepressant fluoxetine, and a third group (NSC, *n* = 22) remained unstressed. Animals were observed in home pens and during dedicated tests to assess resignation and anxiety-like behaviors. Brain structure and function were evaluated *via* proton MRS and fMRI. Hippocampal molecular biology and immunodetection of cellular proliferation (Ki67^+^) and neuron maturation (DCX^+^) in the dentate gyrus were also performed. Salivary cortisol, fecal short-chain fatty acids (SCFAs), and various plasmatic and intestinal biomarkers were analyzed. Compared to NSC, SC animals showed more resignation (*p* = 0.019) and had a higher level of salivary cortisol (*p* = 0.020). SC brain responses to stimulation by a novel odor were lower, similarly to their hippocampal neuronal density (*p* = 0.015), cellular proliferation (*p* = 0.030), and hippocampal levels of BDNF and 5-HT_1A_R (*p* = 0.056 and *p* = 0.007, respectively). However, the number of DCX^+^ cells was higher in the ventral dentate gyrus in this group (*p* = 0.025). In addition, HOMA-IR was also higher (*p* < 0.001) and microbiota fermentation activity was lower (SCFAs, SC/NSC: *p* < 0.01) in SC animals. Fluoxetine partially or totally reversed several of these effects. Exposure to psychosocial stressors in the pig model induced effects consistent with the human and rodent literature, including resignation behavior and alterations of the HPA axis and hippocampus. This model opens the way to innovative translational research exploring the mechanisms of chronic stress and testing intervention strategies with good face validity related to human.

## Introduction

Major depressive disorder is a major cause of disability worldwide [“Depression: let’s talk” says World Health Organization [WHO] (2017)], as depression tops list of causes of ill health. Its commonest symptoms include a depressed mood, anhedonia, and cognitive disorders (Yang et al., 2015). Depression is also highly associated with other mood disorders (e.g., anxiety), coronary, metabolic (e.g., diabetes, obesity) and gastrointestinal disorders (e.g., irritable bowel syndrome) comorbidities (Kennedy et al., 2012; Yang et al., 2015; Joseph and Golden, 2017). One of the leading risk factor for depression is psychological chronic stress (Caspi, 2003), which includes a notion of inescapability and/or uncontrollability (Richter-Levin and Xu, 2018).

In this context, several psychological chronic stress models have been developed in rodents, especially the chronic unpredictable mild stress (CMS) model. CMS consists in a constant exposure to unpredictable micro-stressors (Willner, 2017), which results in the development of several behavioral changes, especially depressive-like (behavioral despair, anhedonia) and anxiety-like behaviors (D’Aquila et al., 1994; Mineur et al., 2006). CMS is also characterized by deregulations of the hypothalamo-pituitary-adrenal (HPA) axis (Goshen et al., 2008), and alterations in specific brain regions (e.g., hippocampus – HPC, prefrontal cortex – PFC) (Banasr et al., 2007; Zhang et al., 2010; Willner, 2017). Chronic stress has also been linked with inflammatory processes (Ramirez et al., 2016; Wohleb and Delpech, 2017), and gut-brain axis deregulations (Cryan and Dinan, 2012, 2015; Dinan et al., 2015; Moloney et al., 2016). Rodent models are widely used in biomedical research. Their simplicity of breeding, feeding and handling has thus permitted a quick development of many strains with a high genetic homogeneity, enabling a low variability and high statistical power. Moreover, their wide use has enabled the quick development of many specific calibrated tests or models related to the different mood disorders. In the field of psychiatric disorders and chronic stress, their use is justified as many symptoms described in patients can be modeled, including anhedonia, behavioral despair, disturbances of neuroendocrine functions and neuroanatomy (decreased hippocampal neurogenesis for instance) (Deussing, 2006). Rodent models have also permitted the validation of many antidepressant molecules. This leads to a relatively good face validity and predictive validity, two of the criteria usually discussed in animal models of psychiatric disorders (Dzirasa and Covington, 2012).

However, rodent models show several limitations regarding the extrapolation of results to human disorders. Because of their size, the amount of biological samples available on each animal is limited. The overall anatomical organization is different between humans and rodents. For instance, which is of particular interest in the case of psychiatric disorders models, the rodent brain is lisencephalic contrary to the gyrencephalic human brain, leading to different structure organization and functioning. Also, their gastrointestinal anatomy, functions and microbiota are very different, due to the body size obviously, but also to dietary habits and life-history traits. Several phyla of human microbiota for instance cannot colonize rodent digestive tract (Pang et al., 2007).

Thus, there is a real need for animal models with a better face validity. Pigs are considered as a good preclinical model for many research and biomedical applications. First, their size allows many investigations with low limitation in terms of samples availability. Their general anatomy, organs size ratio and physiology are comparable to those in humans. Particularly, their brain is also gyrencephalic, and most of the cerebral regions are comparable in terms of structure, vascularization, anatomy, growth, and development (Vodička et al., 2005; Lind et al., 2007). Second, stereotaxic atlases are available, and many classical human imaging techniques have been implemented in the pig model, including functional magnetic resonance imaging (fMRI), computed tomography (CT), single photon emission computed tomography (SPECT), and positron emission tomography (PET) (Clouard et al., 2012b; Roura et al., 2016), which are major investigation tools in the field of mental and neurocognitive disorders. Third, gastrointestinal tracts in pigs and humans also share many similarities in terms of anatomy, morphology, physiology (comparable digesta, transit times, and analogous digestive and absorptive processes), and microbiota composition and activity (Pang et al., 2007; Clouard et al., 2012b; Heinritz et al., 2013). As the microbiota-gut-brain axis is now considered to be deeply involved in neuropsychiatric disorders (Dinan and Cryan, 2016), it is highly relevant to propose an animal model sharing these characteristics with the human.

In this study, we precisely characterized a multifactorial psychological chronic stress model based on a combination of stressors including social isolation, environment impoverishment and unpredictability. The pig is a social animal naturally expressing a large panel of behaviors, and social isolation and/or environmental impoverishment have been shown to impact its behavior and/or physiology (Ruis et al., 1997, 2001; de Jong et al., 1998; Schrader and Ladewig, 1999; Herskin and Jensen, 2000; DeBoer et al., 2015). This first descriptive work was designed to verify that the commonest neurobehavioral symptoms described in patients and rodent models also exist in pigs. To this end, we implemented/adapted several behavioral tests and measures classically used in rodents. In addition, with increasing evidences demonstrating that the microbiota-gut-brain axis is involved in psychiatric disorders, it was important to verify that these types of modulations also exist in this species. Because the pig model provides complementary assets compared to rodent models (in terms of biological sampling opportunity, gut microbiota proximity with the human, individual variability closer to that observed in humans, high-resolution brain imaging, etc.), we chose to perform some microbiota-gut-brain measures that would open the way to further mechanistic explorations, which remain complicated and less relevant in rodents. Performing repeated behavioral and physiological measures over time in pig, in combination with specific *in vivo* and *post mortem* explorations at the gut and brain levels, clearly represents an innovative and original approach in this research scope. Our hypothesis was that our psychosocial chronic stress model might induce depressive symptoms that could be relieved or suppressed *via* the use of the antidepressant fluoxetine.

## Materials and Methods

### Ethics Statement

Experiments were conducted in accordance with the current ethical standards of the European Community (Directive 2010/63/EU), Agreement No. C35-275-32 and Authorization No. 35–88. The Regional Ethics Committee in Animal Experiment of Brittany has validated the entire procedure described in this paper (project numbers 2017070518585877 and 2017080511347475).

### Animal, Housing and Experimental Design

**FIGURE 1:**
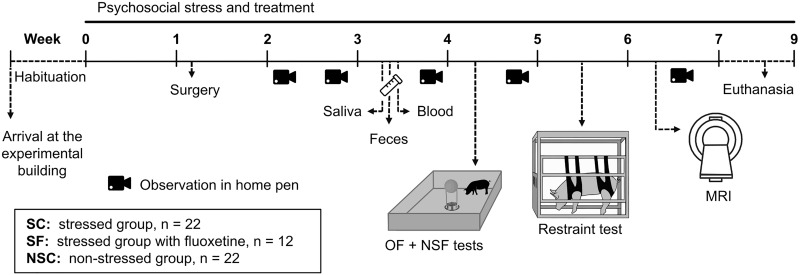
Experimental design of the psychosocial stress, treatment, and behavioral and physiological explorations. At their arrival, pigs were separated into two rooms according to their future group and had 1 week to habituate. They were then subjected to a multifactorial psychosocial stress consisting in social isolation in a poor and unpredictable environment, without (stressed, SC) or with a treatment with the antidepressant fluoxetine (stressed fluoxetine, SF). NSC animals were kept unstressed with pair-housing in an enriched and predictable environment. Behavioral observations were conducted in home pens (Weeks 2, 3, 5, and 6), and during the Openfield (OF), Novelty-Suppressed Feeding (NSF, Week 4), and Restraint (Week 5) tests on 12 animals per group. Pigs were implanted with sensors (Week 1; *n* = 12/group) for continuous measurement of body temperature. Saliva (*n* = 12/group), feces and blood (*n* = 10/group) samples were collected (Week 3). Ten additional females from NSC and SC groups were submitted to a magnetic resonance imaging (MRI) session (Week 6). They were euthanized between Weeks 7 and 9 for further analysis of gut and brain (*n* = 12/group).

Experiments (Figure 1) were carried out from April to June 2017 at the INRA experimental research station of Saint Gilles (France) on fifty-six 65-day-old Piétrain × (Large White/Landrace) pigs. Tails were cut, teeth were clipped, and males were castrated a few days after birth, as usually done in conventional breeding systems. Piglets were housed in conventional farrowing crates until weaning at 28 days and then mixed. At 65 days old they were moved to the experimental building and separated into two rooms according to their group. Twelve animals housed by pairs into double pens (1.75 × 2.65 m) with a same-sex conspecific from their own litter and provided with enrichment (toys) composed the non-stressed group (NSC). Animals in the stressed group (SC, *n* = 12) were housed in individual pens (0.85 × 2.65 m) with an empty pen between each to avoid physical and eye contact, without enrichment and with unpredictable sounds (sirens, metallic noises, gunshots, etc.) and lights randomly diffused (every 10 min ± 30% during the day, every 120 min ± 30% during the night). An additional stressed group received daily 60 mg of Fluoxetine (EG^®^, Laboratoires Eurogenerics, Boulogne-Billancourt Cedex, France) in apple puree (SF, *n* = 12). The three groups were composed of six males and six females. Additional animals (females, NSC and SC, *n* = 10 per group) were necessary for the magnetic resonance imaging (MRI) session, leading to a total of 22 animals in these two groups. Females only were subjected to imaging session to limit variability, as there is an important brain functioning sexual dimorphism that would have necessitated a higher number of animals. To investigate intestinal permeability and brain systems, a second batch of 24 pigs was raised in the same conditions for 8 weeks (NSC and SC, *n* = 12, 50/50 male/female ratio) from October to December 2017. All animals were fed daily with a standard pelleted diet for growing pigs, had unlimited access to water, and were regularly provided with straw. They were subjected to a natural day/night cycle.

### Behavioral Observations

#### Behavior in the Home Pens

Pigs’ postures, behaviors and vocalizations were recorded five times in home pens (Supplementary Table 1) with a 1-min scan sampling (every 10 s) (Figure 1). Data were averaged for the five observations and analyzed *via* a Principal Component Analysis on the FactoMineR plugin of R commander.

#### Openfield (OF) and Novelty-Suppressed Feeding (NSF) Tests

They were conducted to study locomotion in a non-familiar environment and anxiety-like behavior, respectively (Bodnoff et al., 1988; Seibenhener and Wooten, 2015; Blasco-Serra et al., 2017). Pigs were individually led to the waiting area adjacent to the arena (3.5 × 3.5 m). The door was opened and OF started when the animal’s shoulders entered the arena. A camera positioned above the arena enabled the recording of animals’ behavior (Supplementary Table 2) during the 10-min period, using The Observer XT 10 (Noldus, Wageningen, Netherlands). Pigs were led back to the waiting area before introducing food surmounted by a new object (yellow balloon) suspended 60 cm above the trough in the center of the arena. After the pig entered the maze for the second time, the latency to eat was recorded.

#### Restraint Test

The test was adapted from the forced-swim and tail suspension tests that are commonly used in rodents to study resignation as a depression-like symptom, which is usually reversed by antidepressants (Porsolt et al., 1977; Steru et al., 1985; David et al., 2009). Pigs were led to the test device consisting in a homemade metal cage equipped with two suspension harnesses and elevated with an electric system. The test started when the animal’s feet rose off the ground, and the number of attempts to escape and total duration of mobility were recorded for 5 min. A perseverance index was determined as the average duration of one attempt.

All behavioral tests were performed on overnight-fasted animals in a dedicated room.

### MRI Imaging

#### Functional MRI (fMRI) Procedure

The procedure was performed as previously described in Coquery et al. (2018). Due to the anatomical presence of an air cavity behind the skull, a loss of magnetic resonance (MR) signal was detected in some parts of the frontal lobes of several animals. This part of the brain was thus excluded from the analysis and is depicted as a dark gray area on the brain activation maps (cf. fMRI results).

#### Magnetic Resonance Spectroscopy (MRS)

The procedure was performed using the point resolved spectroscopy (PRESS) sequence, with a 30-ms echo time, 1500-ms repetition time, 1000-Hz bandwidth, 1024 readout points, 64 averages, and 1 cm × 1 cm × 1 cm size for the voxel-of-interest (VOI). Two spectra, one of a VOI located in the hippocampus and another of a VOI in the prefrontal cortex, were acquired in each pig. *N*-Acetylaspartate (NAA, 2.0 ppm) and Choline (Cho, 3.2 ppm) were measured, as the NAA/Cho ratio is generally used as a marker of neuronal density (Rudkin and Arnold, 1999; Xia et al., 2016).

#### Olfactory Stimulation

The stimulation was performed with an improved custom-made stimulation apparatus quite similar to that used in Clouard et al. (2012a) on animals equipped with a tube into the right nostril. Each animal was subjected to one block of stimulation/acquisition: odorized stimulation (30 s, 4 L/min) followed by control stimulation (30 s, 4 L/min), repeated eight times. The odorant had never been encountered by pigs before, was mainly composed of *Citrus sinensis* (60–80%) in a vehicle composed of distillated water (60–80%) and glyceryl polyethylene glycol ricinoleate (20–40%), and was provided by Phodé (Terssac, France). For odorized stimulation, the odorant was diluted in distillated water (1 L) at final concentration of 0.105% (w/w), a concentration that was already shown to trigger sensory, hedonic, and cognitive brain responses in anesthetized pigs subjected to the same conditions (Val-Laillet et al., 2016). The control solution consisted in the vehicle diluted in distillated water at a final concentration of 0.2% (w/w).

#### Data and Statistical Image Analyses

##### fMRI

Data analysis was performed with SPM12 (version 6906, Wellcome Department of Cognitive Neurology, United Kingdom). After slice timing correction, realignment and spatial normalization on a pig brain atlas (Saikali et al., 2010), images were smoothed with a Gaussian kernel of 4 mm.

##### Voxel-based statistics

First-level (within-individual contrast) and second-level (within-group contrast) statistics were assessed with a threshold set at *p* < 0.05 to produce the brain maps of activation.

##### Single voxel correction-based statistics

Anatomical regions of interest (ROIs) (Saikali et al., 2010) were used for SVC-based statistics with a *p*-value corrected with a Bonferroni correction at a threshold of 0.05 (peak level). Twelve ROIs corresponding to six bilateral brain structures previously studied (Val-Laillet et al., 2016) were used: hippocampus, amygdala, anterior and dorsolateral PFC, ventral and dorsal anterior cingulate cortex. The related uncorrected *p*-value threshold after Bonferroni correction was 0.0042, which corresponded to a corrected *p*-value of 0.05. Uncorrected *p*-values between 0.0042 and 0.005 were considered as a statistical trend. For voxel- and SVC-based statistics, no suprathreshold voxels were detected with false discovery rate-correction at *p* < 0.05. Due to technical problems during functional acquisition, only 16 out of the 20 animals were used for analysis.

##### MRS

Magnetic resonance spectra were analyzed with the LCModel (Provencher, 1993), using a basis-set provided by the vendor.

### Biological Sampling and Quantitative Analyses

Saliva and blood samples were obtained at 8:30 am in Week 3 on overnight-fasted animals.

#### Salivary Samples

Pigs had to chew cotton buds (Salivette^®^, Sarstedt, Nümbrecht, Germany) for 1 min. Buds were rapidly centrifuged (2,500 × *g*, 10 min, 4°C), and supernatants stored at −20°C. Cortisol was quantified with a luminescence immunoassay kit (IBL, Hamburg, Germany) and read with a luminometer (Mitras LB940 Bertold Technologies).

#### Feces Samples

Samples were collected rectally and processed as described in Lemaire et al. (2018) for quantification of short chain fatty acids (SCFAs) until gas chromatography analysis (Jouany et al., 1981).

#### Blood Samples

Samples were collected from the jugular vein into EDTA, heparin (Vacutest Kima, Arzergrande, Italy) or aprotinin (BD Life Science, Le Pont-de-Claix Cedex, France) vacutainers. Blood from one of the EDTA vacutainers was directly transferred into a tube containing anti-DPP IV (10 μl/ml of blood; Merck, Darmstadt, Germany). Samples were kept in ice until centrifugation (2,500 × *g*, 10 min, 4°C) and plasma was stored at −20°C or −80°C until further analyses. Glucose, NEFA, TG, total cholesterol, HDL-cholesterol, haptoglobin and alkaline phosphatase levels were assessed by an automated spectrophotometric method (Konelab 20i, Thermo Fisher Scientific, Illkirch, France) using specific kits (glucose, TG, total and HDL-cholesterol and alkaline phosphatase: Thermo Fisher Scientific, NEFA: Wako chemicals, France, haptoglobin: Eurobio, Les Ulis, France). Insulin concentrations were obtained using radioimmunoassay kits (EMD Millipore, Billerica, MA, United States). LPS, GLP-1, and PYY levels were measured using ELISA kits (Mybiosource, San Diego, CA, United States; Millipore Corporation, San Diego, CA, United States; Phoenix Pharmaceuticals Inc., Burlingame, CA, United States). Fluoxetine and norfluoxetine levels were analyzed by Liquid Chromatography High Resolution Mass Spectrometry (LC-HR-MS). Plasma samples (200 μL) were extracted by liquid-liquid extraction and analyses were performed by a LC-ESI-HR-MS system equipped with a Thermo Fisher Scientific Q Exactive^TM^ (San Jose, CA, United States) mass spectrometer system as previously described (Boumrah et al., 2016; Gicquel et al., 2016). They were quantified on human plasma-calibration curves using exact mass, respectively, 310.1413 and 296.1257 m/z.

HOMA-IR index evaluating the insulin resistance (Song et al., 2007) was calculated from levels of fasting glucose (mmol/ml) and insulin (μIU/ml) using the formula $\frac{{\mathrm{Insulin}}^{*}\,\mathrm{Glucose}}{22.5}$.

#### Post Mortem Sampling

Pigs were euthanized in the morning of Weeks 7 to 9 in the experimental slaughterhouse by electrical stunning immediately followed by exsanguination. Blood was collected in sterile BD vacutainer^®^ CPT^*TM*^ tubes (BD Biosciences, Le Pont-de-Claix, France) for isolation of peripheral blood mononuclear cells (PBMC). Brains were rapidly PBS-perfused via the carotids. Left hippocampus and PFC striatum were dissected and transported in MACS^®^ Tissue storage solution (Miltenyi Biotech, Paris, France) for immunological studies. The right hemisphere was fixed in 4% paraformaldehyde (48 h, 4°C) and stored (30% sucrose, 0.1% sodium azide, 4°C) for histological analyses. Adrenal glands were dissected and weighed. Segments of jejunum and colon were collected and stored in cold DMEM (Thermo Fisher Scientific, Waltham, MA, United States) for Using chamber measurements, or fixed in 4% PFA (72 h, 4°C) and stored (70% ethanol, 4°C) for histological analyses.

### Immune Cell Analyses

#### Immune Cell Isolation

Purified PBMC were collected at the interface of centrifuged vacutainers (1,500 × *g*, 20 min without brake). Isolation of immune brain mononuclear cells (BMC) was adapted from LaFrance-Corey and Howe (2011).

#### BMC and PBMC Stimulation

Immune cells were suspended and cultured in RPMI 1640 medium (Sigma, St Quentin Fallavier, France) supplemented with 10% fetal calf serum (FCS), 100 IU/ml penicillin and 100 μg/ml streptomycin to achieve cell concentration of 0.5 × 10^6^ cells/ml for BMC in 24-well flat-bottomed plates and 5 × 10^6^ cells/ml for PBMC in 96-well flat-bottomed plates. BMC were cultured overnight, and PBMC for 3 days, with or without LPS (0.5 μg/ml and 10 μg/ml, respectively; ultra-pure *Escherichia coli 0111:B4* strain Gram^–^, Invivogen, Toulouse, France) at 37°C in 5% CO_2_ atmosphere. Culture supernatants of both cell types were stored at −20°C until cytokine quantification. BMC were re-suspended in FCS 10% DMSO (Hybri-max, Sigma) and stored at −150°C until flow cytometry analysis.

#### Cytokine Patterns of BMC and PBMC

Concentrations of TNF-α, IL-1β, and IL-10 were measured in culture supernatants using porcine ELISA kits (R&D Systems, Lille, France). For each measure a ROUT test was performed to exclude animals from the analysis, if relevant.

#### Flow Cytometry Analysis

The analysis was performed on thawed BMC using monoclonal antibodies (mAbs) or isotype-matched mAb as controls. Cells were incubated (20 min, 4°C) with primary mAb recognizing porcine CD11R1 (CD11b, mouse IgG1, AbD Serotec, Colmar, France) and then with goat anti-mouse IgG1-PE (20 min, 4°C). Finally, BMC were stained (20 min, 4°C) with pig-CD45-FITC (mouse IgG1, *BIO-RAD*, Marnes-La-Coquette, France) and analyzed with a MACSQuant analyzer (Miltenyi Biotec, France) equipped with MACSQuantify software.

### Jejunum and Colon Analyses

#### Permeability

After sampling, tissues processing and measurements in Using chamber were performed as described in Hamilton et al. (2015). Paracellular and transcellular permeability were, respectively, measured as the flux of FITC-Dextran (FD-4; Sigma-Aldrich, Saint-Louis, MO, United States), and horseradish peroxidase (HRP Type VI-a; *Sigma-Aldrich*).

#### Morphometry

Samples were embedded in paraffin, cut in sections of 7 μm, and were stained with alcian blue *(Sigma)* and periodic acid Schiff (VWR, Fontenay-sous-Bois, France). They were examined under a light microscope (ApoTome 2, Zeiss, Oberkochen, Germany). Crypts characteristics were assessed in colon and jejunum as well as villi characteristics in jejunum. The analysis was performed on 10 crypts and villi per animal. The number of goblet cells was also counted.

### Brain Molecular Biology

Total RNA was extracted from 100 mg of frozen tissue with phenol/chloroform treatment followed by silica membrane purification (Qiagen, Hilden, Germany) and quantified by a microspectrophotometer (Denovix, DE). 2 μg RNA was converted to cDNA using a High Capacity Complementary DNA Reverse Transcription Kit (Applied Biosystems, Foster City, CA, United States), and RT-PCR was performed with the StepOnePlus real-time PCR machine using Fast SyberGreen master mix *(Applied Biosystems)* for detection. BDNF (Andresen et al., 2009), 5-HT_2B_R (Poletto et al., 2011), and DRD2 (García et al., 2009) pre-existing primers were used. Primers for 5-HT_1A_R (Fwd: GACCTCATGGTGTCAGTGCT; Rev: CACGTAGTCAATGGGGTCTGT) were designed using primer blast. ACTB, YWHAZ, and B2M genes were selected as housekeeping genes. Relative expressions of the target genes were determined using the 2^–ΔΔ*Ct*^ method.

### Brain Immunohistochemistry (IHC)

Thirty-μm brain slices of the right hippocampus were made with a cryomicrotome. Slices were deposited on glass slides and stored at −20°C. Five slices separated from 50 slices, each covering the junction of the ventral and dorsal parts of the hippocampus, were selected for doublecortin (DCX) and Ki67 staining. Both IHC procedures were performed in different but immediately adjacent slices. After being thawed (10 min, ambient temperature) and rinsed three times during 10 min in PBS, 150 μL of blocking buffer (PBS solution, Gibco by Life Technologies; 10% horse serum, Sigma; 0.3% Triton, Sigma) was deposited on the region of interest. Brain slices were stored during 1 h, at ambient temperature in a humidity chamber. Blocking buffer excess was then carefully removed with absorbent tissue, and 150 μL of primary antibody [*Anti-doublecortin (C-18) goat antibody sc8066 1/200, Santa Cruz* or *anti-Ki67 rabbit antibody ab15580 1/200, Abcam*] was deposited on the region of interest. Parafilm was carefully spread on the slice and slides were stored (4°C, 16 h). Slices were then rinsed three times in PBS (10 min, ambient temperature, humidity chamber). After the last rinse, the excess of blocking buffer was removed with absorbent tissue, and 150 μL of secondary antibody *(Cy*^TM^*3-conjugated AffiniPure donkey anti-goat IgG (H+L) 1/500, Jackson ImmunoResearch Laboratories* for DCX, and *Alexa Fluor 488-conjugated anti-rabbit IgG 1/200, Cell Signaling)* was deposited on the slices and incubated (2 h, ambient temperature, humidity chamber). After incubation, the solution excess was carefully removed with absorbent tissue and the slices were rinsed three times with PBS (5 min, ambient temperature). The excess of PBS was then removed. Slides were finally mounted with Fluoroshield Mounting Medium with DAPI (2 drops per slide, *Abcam*) and stored at ambient temperature. Sections were examined under a fluorescence microscope (Eclipse 80i; Nikon, Japan), digitized, and large field mosaics were performed with micro-manager (ImageJ plugin) to get the whole hippocampal section on one image.

GCL volume, number of Ki67^+^ nuclei and of DCX^+^ cells in the GCL were determined as detailed in Val-Laillet et al. (2017). The Cavalieri approach was first used on the five sites for the calculation of total GCL volume and of total number of cells in the GCL. In a second approach, the same analysis was performed site by site, to highlight potential differences along the ventro-dorsal axis. The three first sites were considered as ventral and the two last sites were considered as dorsal hippocampus. Studies investigating the effects of selective lesion of the hippocampus have shown a functional segregation along its dorso-ventral axis (Coquery, 2009). The ventral part was found to be primarily involved in emotion processing (Kjelstrup et al., 2002), presumably due to its preferential connectivity with the amygdala formation (Witter et al., 2000). The dorsal part was found to be more involved in spatial working memory (Pothuizen et al., 2004) with a preferential connectivity with the PFC (Witter et al., 2000).

Two animals with a high number of cells (superior to mean + 2 standard deviation for the totality of the five sections) were excluded from the analyses (one animal for DCX and one for Ki67). For Ki67 counting, one SC animal was also excluded, as the IHC did not work.

### Body Temperature Sensors

At Week 1, pigs were implanted with temperature sensors (Anipill, Caen, France). They were anesthetized by an intramuscular injection of Zoletil^®^ (Virbac, Carros, France) and a 2-cm incision was made on the right neck region at 5 cm below the ear. A sterile temperature logger was implanted into the brachiocephalic muscle at 4 to 5 cm depth. The total duration of the surgical operation did not exceed 10 min. This enabled a continuous measurement of body temperature with accuracy of 0.1°C. This measurement was wirelessly and continuously transmitted to a recorder. Body temperature was measured every 15 min until the end of the experiment, and data from the entire period were pooled and analyzed hour per hour.

### Statistical Analysis

Statistical analyses were performed using SPSS software version 25 *(IBM Corp)*. Comparisons were made with a two-way ANOVA (group × sex × group^*^sex), followed by Fisher’s LSD *post hoc* test when three groups were analyzed. For IHC and BMC data, a one-way ANOVA testing the group factor followed by a LSD test was performed. Residuals were tested for normality with the Shapiro–Wilk test. If they failed to normality (e.g., cortisol levels in saliva), data were log-transformed and the same analysis was performed on transformed data. Data are expressed as mean ± SEM. Differences were considered significant at *p* < 0.05 and as a trend when 0.05 < *p* < 0.1.

## Results

### Behavioral Observations

#### Behavior in Home Pens

**FIGURE 2:**
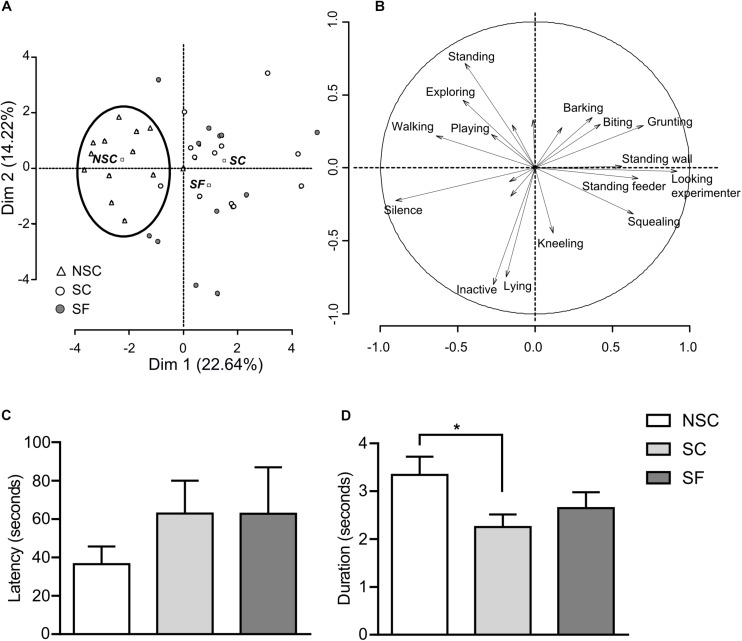
Behavioral assessments. The principal component analysis (PCA) of behaviors expressed by 12 pigs/group in home pens (**A**, animals projections; **B**, behaviors projections) showed that non-stressed (NSC) animals constituted a cluster characterized by the expression of behaviors such as “playing” and “exploring,” while emitting very few vocalizations. Stressed animals treated (SF) or not (SC) with the anti-depressant fluoxetine did not differ and expressed behaviors such as “standing in the feeder,” “looking at the experimenter,” while emitting low- and high-pitched vocalizations. Novelty-suppressed feeding (NSF) test did not show differences in the latency to eat; nevertheless, there was a higher variability in stressed groups **(C)**. *N* = 11–12/group. In restraint test SC had a lower perseverance index than NSC (*p* = 0.019), and SF did not significantly differ from the other groups **(D)**. *N* = 9–11/group, mean ± SEM, two-way ANOVA, ^*^p < 0.05.

Results detailed in Figures 2A,B suggest that stressed and non-stressed animals expressed different behaviors under their living condition, without any detectable effect of fluoxetine. The behavioral variability was also much higher in stressed animals.

#### OF Test

Animals did not show any difference in terms of locomotion measured as duration and number of virtual zones crossed.

#### NSF Test

We did not find any difference at the group level as the variability was important (Figure 2C) but found a trend for a group^*^sex interaction with a higher latency in SC than NSC females [*F*(2,29) = 2.796, *p* = 0.078].

#### Restraint Test

The total duration, number of attempts and number of vocalizations did not differ between groups. However, the perseverance index was significantly lower in SC than in NSC [*F*(1,24) = 2.801, *p* = 0.019]. SF animals had an intermediate perseverance index as they did not differ from SC and NSC (Figure 2D).

### HPA Axis Functioning

**FIGURE 3:**
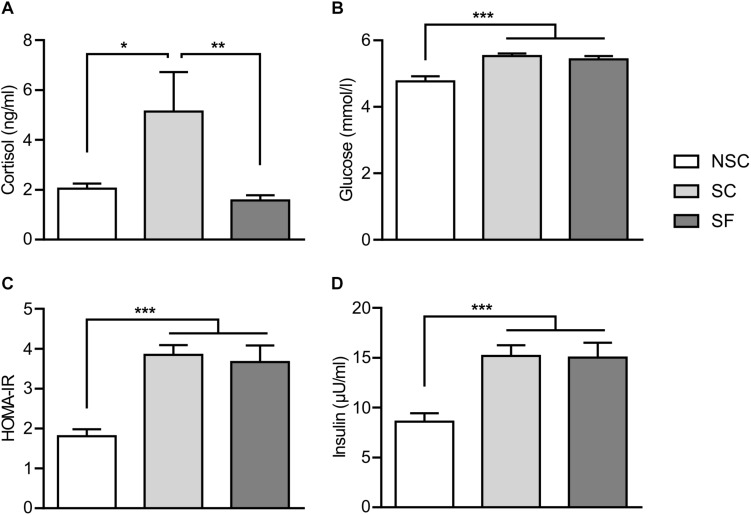
Plasmatic markers. At 3 weeks of stress and treatment, stressed animals (SC) had a higher level of salivary cortisol than non-stressed (NSC) and stressed-treated (SF) animals **(A)** [*F*(2,28) = 6.882, SC/NSC: *p* = 0.20, SC/SF: *p* = 0.001]. *N* = 11/group. The levels of glucose **(B)**, insulin **(D)**, and the insulin resistance index HOMA-IR **(C)** were significantly higher in SC and SF compared to NSC [respectively, *F*(2,23) = 3.429, *F*(2,24) = 9.458, *F*(2,23) = 12.657, NSC/SC, and NSC/SF: *p* < 0.001]. *N* = 10/group, mean ± SEM, two-way ANOVA, ^*^p < 0.05, ^∗∗^*p* < 0.01, and ^∗∗∗^*p* < 0.001.

SC animals had a significantly higher salivary cortisol level than NSC [*F*(2,28) = 6.882, *p* = 0.020] and SF animals (*p* = 0.001) (Figure 3A). As this might indicate a deregulation of HPA axis we investigated the adrenal glands weight ratio but there was no difference.

### Brain Structure and Function

#### Ratio NAA/Cho (Figure 4A)

The NAA/Cho indicator of neuronal density was lower in SC compared to NSC in HPC (*U* = 3.000, *p* = 0.015), but there was no difference in the PFC.

**FIGURE 4:**
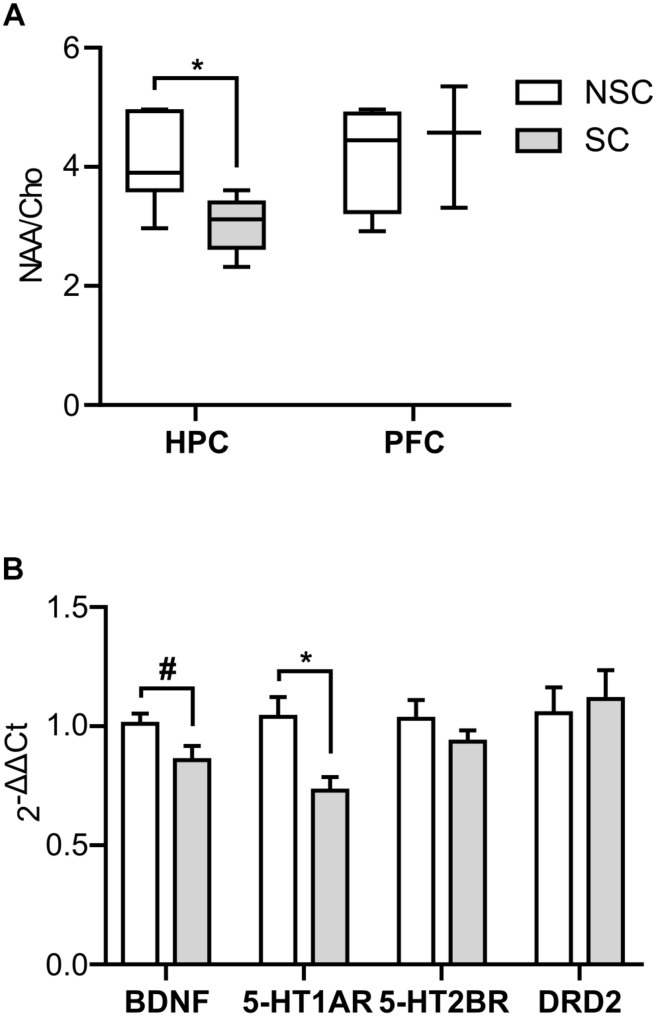
Neurophysiology (single voxel spectroscopy and molecular biology). The NAA/Cho ratio was lower in the hippocampus (HPC) of stressed (SC) compared to non-stressed (NSC) animals (*U* = 3.000, *p* = 0.015), but no difference was found in the prefrontal cortex (PFC) **(A)**. *N* = 6/group (HPC) and *n* = 3/group (PFC), boxplot, Mann–Whitney. In the hippocampus there was a trend for a lower level of BDNF [*F*(1,20) = 4.125, *p* = 0.056] and a significantly lower level of 5-HT_1A_R [*F*(1,18) = 7.959, *p* = 0.007] in SC animals **(B)**. *N* = 12/group, mean ± SEM, two-way ANOVA, ^#^*p* < 0.1, ^*^p < 0.05.

#### BDNF and Monoaminergic Systems

There was a trend for a lower expression level of BDNF in the hippocampus of SC compared to NSC animals [*F*(1,20) = 4.125, *p* = 0.056], and the expression level of 5-HT_1A_ receptor was significantly lower in these animals [*F*(1,18) = 7.959, *p* = 0.007] (Figure 4B). No significant difference was detected in the prefrontal cortex.

#### Immunohistochemistry (Figures 5A–F)

**FIGURE 5:**
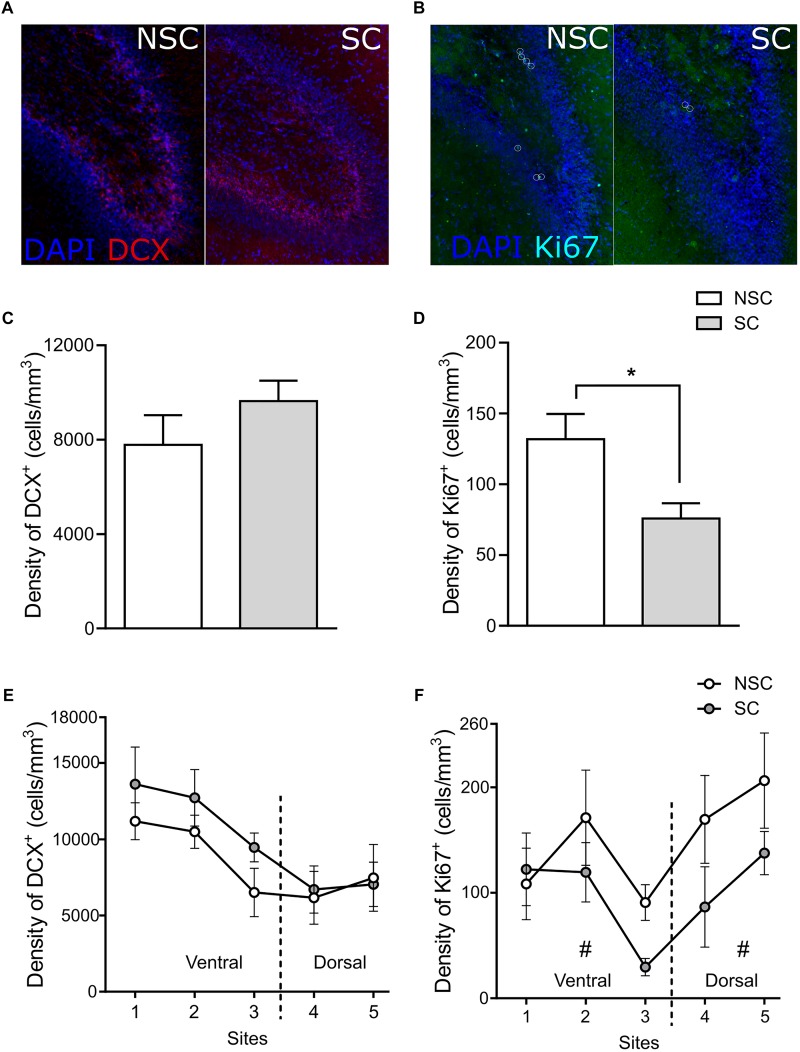
Hippocampal structural plasticity. Distribution of DCX^+^ (doublecortin) **(A)** and Ki67^+^ cells **(B)** in the hippocampus and in the GCL (granular cell layer) of a stressed (SC) and a non-stressed (NSC) animal (DCX/DAPI and Ki67/DAPI markers, respectively). On the totality of the five sections there was no difference in the GCL volume and in the density of DCX^+^ cells in the GCL **(C)**. SC animals had a significantly lower density of Ki67^+^ cells [*F*(1,12) = 6.082, *p* = 0.030] **(D)**. The density of DCX^+^ cells did not differ in any of the observed sites **(E)**, however, the number of cells was significantly higher in the ventral zone of SC compared to NSC animals [*F*(1,12) = 6.535, *p* = 0.025]. The density of Ki67^+^ cells tended to be lower in the ventral and in the dorsal parts of NSC compared to SC animals [*F*(1,12) = 3.604, *p* = 0.082, and *F*(1,12) = 3.678, *p* = 0.079, respectively] **(F)**. *N* = 7–8/group, mean ± SEM, one-way ANOVA, ^#^*p* < 0.1, ^*^p < 0.05.

The Cavalieri analysis showed that there was no difference between groups for the whole GCL volume (NSC: 10.96 ± 0.47 mm^3^, SC: 12.05 ± 0.50 mm^3^) and the density of DCX^+^ cells (NSC: 7782 ± 1260 cells/mm^3^, SC: 9638 ± 866 cells/mm^3^) (Figure 5C). However, SC animals had a lower density of Ki67^+^ cells (7 ± 11 cells/mm^3^) than NSC animals [133 ± 18 cells/mm^3^, *F*(1,12) = 6.082, *p* = 0.030] (Figure 5D).

The second analysis did not highlight difference of volume of the GCL in the ventral (NSC: 7.85 ± 0.42 mm^3^, SC: 8.49 ± 0.37 mm^3^) and in the dorsal (NSC: 3.11 ± 0.24 mm^3^, SC: 3.56 ± 0.37 mm^3^) parts of the hippocampus. In the ventral part, the density of DCX^+^ cells did not differ (NSC: 7783 ± 1260 cells/mm^3^, SC: 9638 ± 866 cells/mm^3^, *p* = 0.127), however, the number of cells was higher in SC than in NSC animals [NSC: 85521 ± 14228 cells, SC: 115664 ± 10646 cells, *F*(1,12) = 6.535, *p* = 0.025]. There was no difference in the number and density of DCX^+^ cells in the dorsal part (Figure 5E). The density of Ki67^+^ cells tended to be lower in SC in both ventral [NSC: 111.5 ± 18.6 cells/mm^3^, SC: 66.2 ± 11.6 cells/mm^3^, *F*(1,12) = 3.604, *p* = 0.082] and dorsal parts [NSC: 187.7 ± 30.9 cells/mm^3^, SC: 107.1 ± 25.3 cells/mm^3^, *F*(1,12) = 3.678, *p* = 0.079] (Figure 5F).

#### Brain Responses to a Non-familiar Odorant (NO) (Figures 6A,B)

The stimulation with NO vs. control yield contrasted responses in the NSC and SC groups. When stimulated with the NO, NSC animals showed a higher activation in the hippocampus and the prepyriform cortex than SC, while SC animals showed a higher activation in the dorsal-anterior cingulate cortex, amygdala, and anterior PFC. The corrected SVC-based statistic showed a higher activation in the right hippocampus of NSC vs. SC animals, and in the dorsal-anterior cingulate cortex and anterior PFC of SC vs. NSC animals.

**FIGURE 6:**
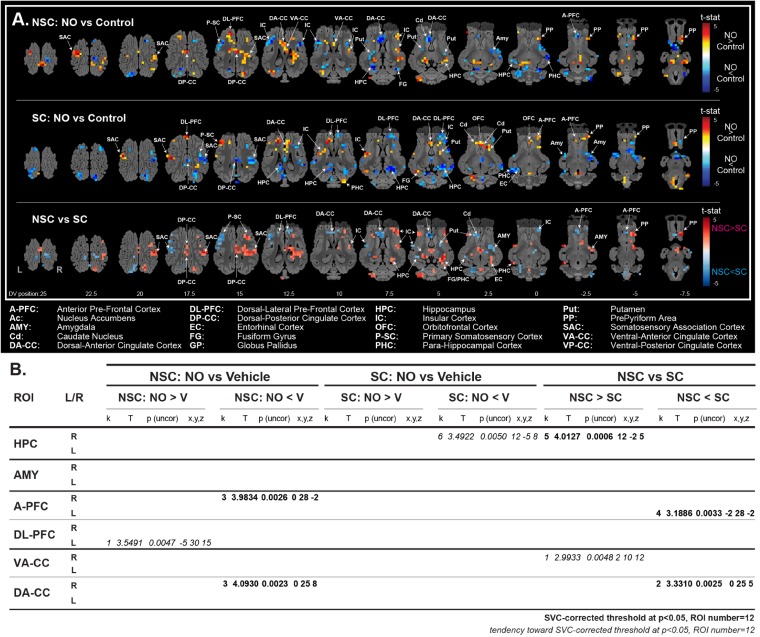
Brain metabolism and function. The BOLD response to the stimulation with a new odorant (NO) was significantly higher in the right HPC and lower in the left A-PFC and right DA-CC of non-stressed (NSC) compared to stressed (SC) animals (**A**, brain activation maps; **B**, coordinates and statistical values of significant clusters). *N* = 8/group, Single Voxel Corrected (SVC) threshold at *p* < 0.05, Regions of interest (ROI) number = 12.

### Energetic Metabolism and Hormonal Gut-Brain Dialogue

Levels of plasmatic glucose, insulin and HOMA-IR differed as detailed in Figures 3B–D. No difference was found in the levels of plasmatic lipids and gut hormones.

### Microbiota Activity

**FIGURE 7:**
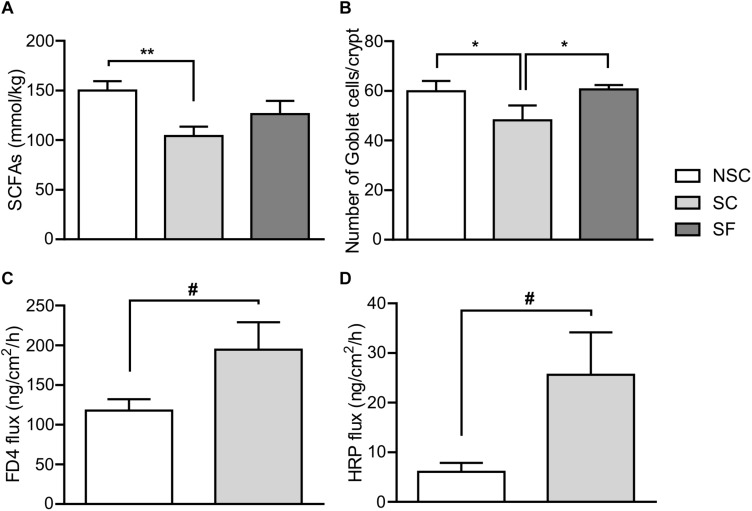
Gut microbiota and physiology. At Week 3 the total quantity of SCFAs **(A)** was significantly lower in stressed (SC) than in non-stressed (NSC) animals [*F*(2,18) = 4.397, *p* = 0.008]. Treated-stressed (SF) animals did not differ from SC and NSC. *N* = 7–9/group. The number of goblet cells per crypt in SC was significantly lower in the jejunum compared to NSC and SF [*F*(2,17) = 7.729, SC/NSC: *p* = 0.011, SC/SF: *p* = 0.008] **(B)**. *N* = 8/group, mean ± SEM, two-way ANOVA. After 8 weeks of stress, permeability tended to be higher in SC compared to NSC: paracellular permeability in colon, *F*(1,20) = 3.956, *p* = 0.061, *n* = 12/group, **(C)**; transcellular permeability in jejunum, *F*(1,19) = 4.311, *p* = 0.052, *n* = 11–12/group, **(D)**, Mean ± SEM, one-way ANOVA, ^#^*p* < 0.1, ^*^p < 0.05, and ^∗∗^*p* < 0.01.

The quantification of total SCFAs in feces highlighted differences between groups (Figure 7A). SC animals had a lower level of acetate [SC: 57.85 ± 5.056 mmol/kg, NSC: 79.95 ± 4.30 mmol/kg, *F*(2,18) = 7.070, *p* = 0.002], isobutyrate [SC: 2.80 ± 0.24 mmol/kg, NSC: 4.38 ± 0.44 mmol/kg, *F*(2,18) = 5.525, *p* = 0.006] and propionate [SC: 22.40 ± 2.23 mmol/kg, NSC: 32.81 ± 2.11 mmol/kg, *F*(2,18) = 0.868, *p* = 0.029] compared to NSC animals. A lower concentration was observed in SF compared to NSC animals for acetate (65.28 ± 3.47 mmol/kg, *p* = 0.021) and isobutyrate (3.01 ± 0.33 mmol/kg, *p* = 0.011).

### Fluoxetine and Norfluoxetine

Fluoxetine and norfluoxetine levels were quantified in plasma ∼24 h after the last medication. The levels found in SF animals were, respectively, 1.26 ± 0.25 μg/l and 6.84 ± 0.90 μg/l. Plasma from SC animals were free of both metabolites.

### Circulating and Brain Immune Cells Analysis

Results at the peripheral and cerebral levels are detailed in Table 1.

**TABLE 1:** Peripheral and brain immunological assessments.

		**Mean ± SEM**	***F***	***p*-value**	
			
		**SC**	**NSC**		
Peripheral level	Number of PBMC (in millions)	28.41 ± 4.88	23.16 ± 2.56	*F*(1,16) = 1.926	0.184
	TNF-α (unstimulated PBMC) (pg/ml)	16.52 ± 8.77	190.12 ± 76.17	*F*(1,16) = 0.034	**0.096**
	TNF-α (LPS-stimulated PBMC) (pg/ml)	723.58 ± 141.74	697.77 ± 131.88	*F*(1,16) = 0.003	0.959
	IL-10 (unstimulated PBMC) (pg/ml)	36.87 ± 14.44	80.59 ± 62.76	*F*(1,16) = 0.440	0.456
	IL-10 (LPS-stimulated PBMC) (pg/ml)	470.68 ± 83.19	598.23 ± 85.99	*F*(1,16) = 0.995	0.333
	TNFα/IL-10 ratio (unstimulated PBMC)	0.79 ± 0.21	0.95 ± 0.24	*F*(1,15) = 1.768	0.689
	TNFα/IL-10 ratio (LPS-stimulated PBMC)	1.61 ± 0.44	1.06 ± 0.28	*F*(1,15) = 0.239	0.632
	LPS in plasma (ng/ml)	22.04 ± 1.11	27.90 ± 1.34	*F*(1,16) = 10.795	**0.005**
	Haptoglobin in plasma (mg/ml)	1.48 ± 0.20	1.66 ± 0.42	*F*(1,16) = 0.160	0.694
	Alkaline phosphatase in plasma (U/L)	19.94 ± 6.30	16.28 ± 5.15	*F*(1,16) = 0.746	0.4
Cerebral level		Hippocampus			
	Number of immune cells/g of tissue (in millions)	8.21 ± 1.28	10.68 ± 0.69	*F*(1,14) = 2.921	0.11
	TNF-α (unstimulated cells) (pg/ml)	105.97 ± 27.08	91.40 ± 18.57	*F*(1,14) = 0.197	0.664
	TNF-α (LPS-stimulated cells) (pg/ml)	944.63 ± 173.05	626.65 ± 114.74	*F*(1,14) = 2.745	0.12
	IL-1β (unstimulated cells) (pg/ml)	147.03 ± 46.95	157.33 ± 26.66	*F*(1,14) = 0.036	0.851
	IL-1β (LPS-stimulated cells) (pg/ml)	1399.49 ± 252.31	1386.76 ± 229.86	*F*(1,14) = 0.005	0.943
	IL-8 (unstimulated cells) (pg/ml)	946.68 ± 241.95	786.93 ± 164.99	*F*(1,14) = 0.298	0.594
	IL-8 (LPS-stimulated cells) (pg/ml)	1399.50 ± 252.31	1386.76 ± 229.86	*F*(1,14) = 1.509	0.24
	BIC/resident cells ratio	0.47 ± 0.21	0.56 ± 0.23	*F*(1,9) = 0.418	0.534
		Prefrontal cortex (PFC)			
	Number of immune cells/g of tissue (in millions)	5.46 ± 0.81	7.79 ± 0.98	*F*(1,14) = 3.363	**0.088**
	TNF-α (unstimulated cells) (pg/ml)	315.04 ± 44.34	223.88 ± 28.55	*F*(1,14) = 2.988	0.106
	TNF-α (LPS-stimulated cells) (pg/ml)	990.27 ± 135.23	801.34 ± 44.06	*F*(1,14) = 0.591	0.455
	IL-1β (unstimulated cells) (pg/ml)	335.58 ± 29.65	238.79 ± 37.51	*F*(1,14) = 4.099	**0.062**
	IL-1β (LPS-stimulated cells) (pg/ml)	1741.64 ± 168.59	1704.69 ± 216.79	*F*(1,14) = 0.048	0.83
	IL-8 (unstimulated cells) (pg/ml)	938.67 ± 145.62	862.71 ± 84	*F*(1,14) = 0.179	0.679
	IL-8 (LPS-stimulated cells) (pg/ml)	1902.96 ± 150.93	1701.73 ± 111.78	*F*(1,14) = 0.738	0.405
	BIC/resident cells ratio	5.57 ± 2.11	3.08 ± 1.09	*F*(1,13) = 1.122	0.309
		Striatum			
	Number of immune cells/g of tissue (in millions)	8.24 ± 1.14	11.88 ± 1.53	*F*(1,14) = 3.614	**0.078**
	TNF-α (unstimulated cells) (pg/ml)	139.84 ± 36.18	65.87 ± 14.27	*F*(1,14) = 3.617	**0.078**
	TNF-α (LPS-stimulated cells) (pg/ml)	523.09 ± 105.77	397.75 ± 72.76	*F*(1,14) = 0.201	0.661
	IL-1β (unstimulated cells) (pg/ml)	168.08 ± 58.71	105.47 ± 20.22	*F*(1,14) = 1.017	0.33
	IL-1β (LPS-stimulated cells) (pg/ml)	1181.80 ± 165.36	1223.34 ± 112.33	*F*(1,14) = 0.286	0.601
	IL-8 (unstimulated cells) (pg/ml)	519.41 ± 131.49	451.17 ± 77.42	*F*(1,14) = 0.200	0.662
	IL-8 (LPS-stimulated cells) (pg/ml)	1642.90 ± 153.91	1476.91 ± 148.94	*F*(1,14) = 0.412	0.532
	BIC/resident cells ratio	*N*/*A*	*N*/*A*	N/A	N/A

In periphery, there was a higher number of peripheral blood mononuclear cells (PBMC) in stressed (SC) than in non-stressed (NSC) females (group^*^sex: *p* = 0.006), suggesting an effect of the psychosocial stress on the recruitment of peripheral immune system in females. There was a trend for a lower level of the pro-inflammatory cytokine TNF-α in unstimulated PBMC in SC, but this difference disappeared when cells were LPS-stimulated. At Week 3, the level of plasmatic lipopolysaccharide (LPS) was lower in SC than NSC animals but there was no difference in haptoglobin and alkaline phosphatase levels (*N* = 8–12/group, two-way ANOVA). At the cerebral level there was no difference in the hippocampus. In the PFC and striatum there was a trend for a lower number of immune cells in SC than NSC. In SC there was also a trend for a higher level of the pro-inflammatory cytokines IL-1β (PFC) and TNF-α (striatum) in unstimulated cells, but these differences disappeared with LPS-stimulation (*N* = 8/group, one-way ANOVA). Thus, without stimulation, brain immune cells from SC animals seemed to be more inflammatory that the ones from NSC, as it is known that chronic stress is linked with such modulation of inflammatory processes (Ramirez et al., 2016; Wohleb and Delpech, 2017). BIC – Brain Infiltrating Cells (*CD11b^+^CD45^*high*^*). Bold values indicate a significant difference between groups or a statistical trend.

### Intestinal Barrier

#### Morphometry (Figure 7B)

There was a lower number of goblet cells in both villi and crypts of the jejunum in SC than NSC animals [in villi, SC: 47.30 ± 5.063, NSC: 60.48 ± 4.83, *F*(2,17) = 2.896, *p* = 0.040], and SF animals did not differ from NSC. There was no difference in the colon.

#### Permeability (Figures 7C,D)

In the colon, there was a trend for an increased permeability to FD-4 in SC animals [*F*(1,20) = 3.956, *p* = 0.061] but no difference in HRP permeability. Jejunal permeability to HRP tended to be higher in SC than in NSC animals [*F*(1,19) = 4,311, *p* = 0.052] but there was no difference in FD-4 permeability.

### Body Temperature

**FIGURE 8:**
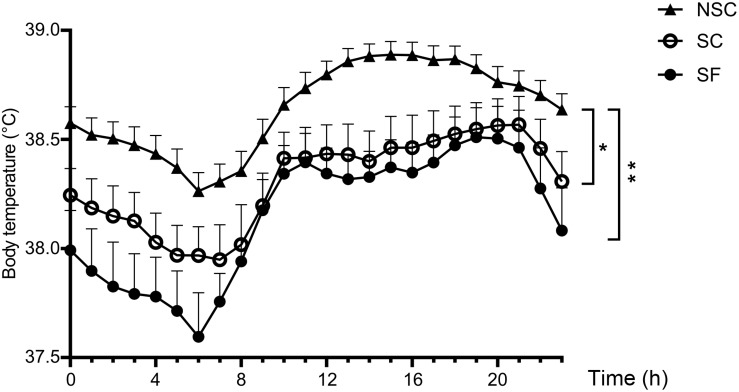
Body temperature. Body temperature was significantly higher in non-stressed (NSC) than in stressed (SF and SC) animals. *N* = 12/group, mean ± SEM, two-way ANOVA, ^*^p < 0.05, ^∗∗^*p* < 0.01.

The averaged body temperature was lower in SC and SF compared to NSC [SC: 38.30 ± 0.13 °C, SF: 38.15 ± 0.14°C, NSC: 38.64 ± 0.06°C, *F*(1,30) = 4.762, SC/NSC: *p* = 0.047 and SF/NSC: *p* = 0.005]. The differences varied along time during day and night as presented in Figure 8.

## Discussion

The first aim of this study was to validate a psychosocial chronic stress model combining several stressors in a pig model. We measured parameters classically depicted in rodent chronic stress models and in human patients, and extended our investigations to the gut physiology and microbiota activity, as well as to functional brain responses to sensory stimulation.

### Chronic Stress, Brain, and Behavior

Freely expressed behaviors differed in home pens between groups. Non-stressed (NSC) animals expressed more play and exploration behaviors considered as positive in terms of welfare (Reimert et al., 2013), whereas stressed (SC) animals emitted more vocalizations and looked more at the experimenter, what we interpreted as a social incentive to the human to palliate the absence of a pen mate. Stressed animals also demonstrated a higher variability in the NSF test, and females from the SC group showed a trend for a higher latency to feed, suggesting limited impacts of our model on anxiety. Moreover, SC animals showed a lower perseverance index, which might suggest an inclination toward resignation, which is a core indicator in animal models of depression (Porsolt et al., 1977; Steru et al., 1985; David et al., 2009). The experimental constraints did not allow us to investigate anhedonia, another important component of depressive behavior (Treadway and Zald, 2011; Zielinski et al., 2017) that might be further tested in a sucrose-preference test for example. Though, during the olfactory stimulation paradigm in fMRI, the new odor induced different activation in brain areas that are part of the corticolimbic reward circuit in SC compared to NSC animals. Further work is needed to investigate whether our chronic stress model alters the susceptibility of animals to experience pleasure and demonstrate hedonic motivation.

The HPA axis acts as a key regulator of the stress response, and appears to be deregulated in the context of depression, leading to high levels of circulating glucocorticoids (Goshen et al., 2008; Taylor and Fink, 2008). Salivary levels of cortisol, the major glucocorticoid in pigs and humans, were higher in SC animals and suggested a deregulation of the HPA axis. Since cortisol receptors are ubiquitous (Ballard et al., 1974), higher levels of cortisol might lead to various deleterious effects. The assumed lower neuronal density and responses to a new sensory stimulation in these animals likely indicated structural and functional alterations in the hippocampus, which is particularly sensitive to glucocorticoids (Dranovsky and Hen, 2006; Nasca et al., 2015). The effect was particularly visible during the perception of a new odorant in fMRI, with a significantly decreased brain response in the hippocampus of SC animals. The inhibitory effect of glucocorticoids on hippocampal neurogenesis has been widely reported in studies including rodents (Santarelli et al., 2003; Crochemore et al., 2005; Goshen et al., 2008; David et al., 2009; Quesseveur et al., 2013; Kreisel et al., 2014) and seems verified also in our model as there were a lower cell proliferation (expressed as density of Ki67^+^ cells) and level of BDNF in SC animals. Lower levels of 5-HT_1A_R in SC animals were also expected since the serotoninergic system is particularly impacted in the context of depression (Savitz et al., 2009), and several fluoxetine effects appear to be dependent on the activation of this receptor (Santarelli et al., 2003). Surprisingly, the higher number of DCX^+^ cells in the ventral hippocampus suggests a higher number of maturating neurons in SC animals. Studies have shown a functional segregation of the hippocampus along its dorsal-ventral axis (Coquery, 2009; Anacker and Hen, 2017), and the ventral part seems to be particularly involved in emotional behavior, social interactions and stress resilience (Anacker and Hen, 2017). This might indicate that, in this specific zone, the DCX^+^ cells did not differentiate into mature and functionally integrated neurons, which might be later subjected to an apoptotic mechanism. Further work investigating the number and organization of mature neurons might answer this question. Another study also reported an increase of DCX^+^ cells in CMS (Wolf et al., 2018), but did not investigate the number and/or functionality of mature neurons.

Fluoxetine partially or totally reversed several criteria including the hypercortisolemia and resignation behavior, but some effects only appeared as statistical trends. Plasmatic concentrations of fluoxetine and norfluoxetine were lower than in treated patients (Amsterdam et al., 1997) and might not be sufficient in this species. As no study had investigated the effect of fluoxetine in pigs before, we used the human and dog posology, but the pharmacokinetics should be further studied to ensure an optimal effect.

### Microbiota-Gut-Brain Axis Implications

The involvement of microbiota-gut-brain axis in neuropsychiatric disorders has recently been investigated in numerous studies (Cryan and Dinan, 2012, 2015; Dinan et al., 2015; Moloney et al., 2016; Valles-Colomer et al., 2019), as understanding these relationships could shed light on their metabolic and gastrointestinal comorbidities. SC animals exhibited a higher HOMA-IR, which constitutes a predisposition to type-2 diabetes (Tang et al., 2015) and could be an early indicator of the onset of metabolic disorders (Shomaker et al., 2011; Hasan et al., 2014). The gut anatomy and barrier function were also altered in SC animals, with fewer goblet cells and an increased paracellular and transcellular permeability. These alterations of the gut barrier might result in an increased influx of pro-inflammatory molecules from the lumen such as LPS, and in visceral pain, which is a frequent symptom of depression (Lackner et al., 2004; Maes et al., 2012). An altered microbial composition has been reported in rodent models and depressive patients (Bailey et al., 2011; Bharwani et al., 2016; Dunphy-Doherty et al., 2018), and microbiota manipulations by fecal transplantation or pre/probiotics are able to improve behavioral and physiological conditions (Dinan et al., 2013; Sarkar et al., 2016). The lower microbiota activity observed in SC animals might be due to a lower microbiota diversity or metabolic activity, and on-going sequencing data will help disentangling this question. SCFAs play a regulatory role in many processes (Koh et al., 2016) including brain functional responses and neurogenesis (Val-Laillet et al., 2018). Interestingly, a butyrate oral supplementation in growing pigs was found to increase hippocampal neurogenesis and modulate brain activity in the hippocampus and striatum. Lower levels of SCFAs might be linked similarly to neurophysiological and behavioral manifestations. The impact of fluoxetine on microbiota has not been well documented yet but would deserve more attention. The high level of systemic cortisol might be the common link between the observed effects, as it is susceptible to impact hippocampal structure and neurogenesis (Goshen et al., 2008; Kvarta et al., 2015), carbohydrates metabolism (Vogelzangs et al., 2007; Joseph and Golden, 2017) and gut and microbiota structure and function (Amini-Khoei et al., 2019). Our results suggest a limited impact on the immune system. However, as chronic low-grade inflammation is often reported in depressive patients (Felger and Lotrich, 2013; Rohleder, 2014; Zalli et al., 2016; Leonard, 2018), it might be interesting to pursue this investigation in more details. The link between body temperature and depressive states is not well understood yet (Pflug et al., 1976; Rausch et al., 2003), and the difference observed in our study is difficult to interpret, as it could be due to housing conditions (i.e., social isolation vs. pairs).

### Variability Impact

There is a high variability in the way to respond to a similar stressor (Sapolsky, 1994; Willner, 2017) and to antidepressant drugs (Al-Harbi, 2012; Alboni et al., 2017; Kokras and Dalla, 2017). Women for example are at higher risk to develop depression while men are more subjected to alcohol dependence (World Health Organization [WHO], 2019, Gender and women’s mental health). Gender is reported as a primordial criterion in stress responses but was only sparsely found in our study, suggesting a limited impact of sex in our model. This might be due to the fact that animals were relatively young, and that males were castrated, which reduced the effects of sexual hormones. Rodent studies usually increase statistical robustness by including only animals from one sex and of high genetic homogeneity for the study of highly complex mechanisms. The genetic heterogeneity of our crossbred pigs also increased variability and thus decreased statistical robustness, but offers a better picture of the heterogeneity of human populations. Indeed a good animal model should illustrate sufficient inter-individual variability as observed in humans, to work on the development of personalized therapeutic strategies.

## Conclusion

Our model induced the onset of depression-like behavior associated with significant alterations of the HPA axis, hippocampus and microbiota-gut-brain axis, with several aspects reversed by the antidepressant treatment. As this first study was quite descriptive, further work should be conducted to elucidate the mechanisms underlying these effects and to explore the correlations and causal relationships between parameters such as cortisol and microbiota. Moreover, the pig is physiologically very close to humans (Clouard et al., 2012b; Roura et al., 2016), leading to a particularly good face validity concerning depressive states. In conclusion, this model represents a novel asset to contribute to fundamental but also translational research, for understanding mechanisms as well as testing new therapeutic and preventive strategies.

## Data Availability

The raw data supporting the conclusions of this manuscript will be made available by the authors, without undue reservation, to any qualified researcher.

## Ethics Statement

Experiments were conducted in accordance with the current ethical standards of the European Community (Directive 2010/63/EU), Agreement No. C35-275-32 and Authorization No. 35–88. The Regional Ethics Committee in Animal Experiment of Brittany has validated the entire procedure described in this manuscript (project numbers 2017070518585877 and 2017080511347475).

## Author Contributions

DV-L, NC, PE, VN, and SoM designed the research. SoM, SaM, SF-B, SG, VR, LLN, and GR performed the research. SoM, SaM, SF-B, and NC analyzed the data. SF-B, SG, VR, LLN, GR, and GG contributed new reagents or analytic tools. SoM wrote the manuscript and all authors read and revised the manuscript.

## Conflict of Interest Statement

SoM, VN, and PE are employees of Phodé. The remaining authors declare that the research was conducted in the absence of any commercial or financial relationships that could be construed as a potential conflict of interest.
