# Supplementary material for: Validation of a Psychosocial Chronic Stress Model in the Pig Using a Multidisciplinary Approach at the Gut-Brain and Behavior Levels
Source: Front Behav Neurosci. 2019 Jul 16;13:161. doi: 10.3389/fnbeh.2019.00161 (PMC6646532; doi:10.3389/fnbeh.2019.00161)
Supplement: Supplementary file 1 [file Table_1.DOCX]

**Validation of a psychosocial chronic stress model in the pig using a multidisciplinary approach at the gut-brain and behavior levels**

**Supplemental materials and methods**

**Table 1. Ethogram used for observations in home pens**

| Category | Behavior | Description |
| --- | --- | --- |
| Posture | Standing | Pig standing or walking on its four legs |
|  | Standing in the feeder | Fore legs alone or fore and hind legs in the feeder |
|  | Sitting | Pig standing on fore legs, hind quarter on the floor |
|  | Kneeling | Pig standing on hind legs, knees on the floor |
|  | Lying | Pig lying on side or sternum |
|  | Standing against the wall | Pig standing on hind legs, fore legs against the wall |
| Activity | Freezing | Pig standing and doesn't move, head down in the shoulders |
|  | Self-explanatory | Pig cleaning or scratching itself with a part of its own body |
|  | Exploring | Pig sniffing, liking, rubbing its snout on the ground or on the wall |
|  | Inactive | Pig not showing any activity |
|  | Walking | Pig walking through the pen |
|  | Looking at the experimenter | Pig looking at the experimenter |
|  | Biting | Pig chewing or biting a bar of its home pen |
|  | Playing | Pig running and moving its head and hind |
|  | Maintenance | Pig defecating or urinating |
|  | Eating/drinking | Pig eating in the feeder or using the water nipple to obtain water |
|  | Scratching | Pig scratching itself against the wall |
| Vocalization | Squealing | High-pitched vocalizations |
|  | Grunting | Low-pitched vocalizations |
|  | Barking | Low tone vocalizations that sound like “woof” (Reimert et al., 2013) |
|  | Silence | No vocalization |

**Table 2. Ethogram used for Openfield analysis**

| Category | Behavior | Description |
| --- | --- | --- |
| Posture | Standing | Pig standing or walking on its four legs |
|  | Sitting | Pig standing on fore legs, hind quarter on the floor |
|  | Kneeling | Pig standing on hind legs, fore legs on the floor |
|  | Lying | Pig lying on side or sternum |
|  | Standing against the wall | Pig standing on hind legs, fore legs against the wall |
| Activity | Freezing | Pig standing and doesn't move, head down in the shoulders |
|  | Self-explanatory | Pig is cleaning itself or scratching with a part of its own body |
|  | Exploring the ground | Pig sniffing, liking, rubbing its snout on the ground |
|  | Exploring the wall | Pig sniffing, liking, rubbing its snout on the wall |
|  | Inactive | Pig not showing any activity |
|  | Walking | Pig walking |
|  | Running | Pig running |
|  | Attempt to escape | Pig trying to jump above the wall |
|  | Playing | Pig running and moving its head and hind |
|  | Maintenance | Pig defecating or urinating |
|  | Scratching | Pig scratching itself against the wall |
| Vocalization | Squealing | High-pitched vocalizations |
|  | Grunting | Low-pitched vocalizations |

**References**

Reimert, I., Bolhuis, J. E., Kemp, B., and Rodenburg, T. B. (2013). Indicators of positive and negative emotions and emotional contagion in pigs. *Physiol. Behav.* 109, 42–50. doi:10.1016/j.physbeh.2012.11.002.
